# Examination of Spectral Properties of Medicinal Plant Leaves Grown in Different Lighting Conditions Based on Mint Cultivation

**DOI:** 10.3390/s21124122

**Published:** 2021-06-15

**Authors:** Mateusz Feldzensztajn, Paweł Wierzba, Adam Mazikowski

**Affiliations:** 1 Department of Metrology and Optoelectronics, Faculty of Electronics, Telecommunications and Informatics, Gdansk University of Technology, 11/12 Narutowicza Street, 80-233 Gdansk, Poland; pwierzba@eti.pg.edu.pl; 2 NIVISS Sp. z o. o. Sp. k., Rdestowa Street 53D, 81-577 Gdynia, Poland

**Keywords:** spectral measurements, colorimetric analysis, horticultural lighting, measurement system, controlled environmental conditions, hydroponic cultivation

## Abstract

Cultivation in controlled environmental conditions can provide good quality medicinal herbs with consistent properties. A sensing system that can determine the contents of medicinal substances in plants using spectral characteristics of leaves would be a valuable tool. Viability of such sensing approach for mint had to be confirmed experimentally, as no data correlating contents of medicinal substances with spectral characteristics of leaves are available, to the best of authors’ knowledge. In the first stage, presented in this paper, the influence of lighting on mint (Mentha rotundifolia) grown on a small hydroponic plantation was studied. Spectral characteristics of leaves were recorded by a spectrophotometer and colorimetric analysis was used to investigate the relationship between these characteristics and the spectrum of lighting. Dry mass yield was measured to test its dependence on the lighting. Dependence of chromaticity of leaves on the spectrum of light used in the cultivation was confirmed. Averaged spectra of leaves are distinguishable using a spectrophotometer and—in most cases—by a human observer. A partial correlation is observed between dry mass yield and the spectrum of lighting. Obtained results justify further research into the correlation between lighting and the contents of biological substances in medicinal plants using spectral characteristics of leaves.

## 1. Introduction

The use of herb-derived compounds in medicine and preventive healthcare is common, while the scope of use of some substances is steadily increasing. One of the most commonly used medicinal herbs is mint, due to its broad range of applications. It occurs in several varieties, the most common of which is peppermint, or round-leaved mint (*Mentha rotundifolia*). Mint leaves and herbs contain compounds with broad biological activity [1,2,3]. Menthol is the primary compound of the essential oil of peppermint (55%). Other compounds of the peppermint oil are limonene, cineole, menthone, menthofuran, isomenthone, menthyl acetate, isopulegol, pulegone and carvone [3].

The oil of mint has been used for medical purposes since antiquity, mostly to treat headache, common cold and neuralgia [3,4,5,6]. It can also soothe skin irritations and gastrointestinal problems and has anti-spasmolytic effects. Moreover, the food processing and flavoring industry has shown a growing interest in some compounds present in mint, in their campaign to replace synthetic preservatives and flavors with substances of plant-origin [3].

The herbal raw material for mint is herb, in particular leaves. As with other herbs, they are harvested mainly from the natural environment. However, it may cause problems that are difficult to control. Firstly, the composition of the obtained oil depends on the environmental conditions, e.g., sunlight, as well as on genetic and phenotypic variability of the plant. Secondly, the drying process and dried plant storage conditions can have an adverse impact on medicinal properties of the material [7]. This is particularly important in the case of obtaining raw material only for a limited period of time during the year. Finally, intensive harvesting of raw material causes the destruction of natural habitats [8].

All the aforementioned problems can be addressed by employing local cultivation in controlled environmental conditions. Growing good quality medicinal plants in a repeatable way requires proper conditions, in particular a suitable illumination. Use of artificial lighting fits well in the need to ensure repeatable conditions for plant growing.

For the last few years, artificial lighting systems have been playing an increasingly important role in plant cultivation [9,10,11,12], due to the remarkable progress of lighting technology. However, the behavior of plants, e.g., flowering [13] and rooting [14], as well as the color of leaves [15,16], depend on the spectrum of light used in the cultivation process. The contents of certain medicinal substances, such as THC in plants (i.e., cannabis) also depends on that spectrum [17], making the selection of appropriate lighting an important issue in the cultivation process. This selection is difficult to perform, especially for medicinal plants, due to scarcity of data relating lighting to contents of the substances of interest.

At present, the content of medicinal substances in plants is most often determined using chemical analysis. This method is destructive, labor-intensive and time-consuming, requiring acquisition of plant matter, delivery to a laboratory, extensive sample preparation and several analyses. A sensing system that could determine the contents of medicinal substances in plants in situ and, preferably, in vivo would be a valuable tool. Acquisition of spectra of leaves and analysis of their properties is one of the most promising sensing methods for such a system. Viability of this method, however, has to be determined experimentally, as the published data regarding variability of such spectra in mint are not available, to the best of the authors’ knowledge.

Therefore, in order to perform such a determination, research using round-leaved mint (*Mentha rotundifolia*) has been undertaken. The research process was divided into two stages. The first stage covered investigation of spectral characteristics of leaves and their variability induced by lighting. Successful completion of this work is a necessary condition for initiating the second stage of research in the future. In the second stage, the relationship between the spectral properties of leaves and chemical composition of mint oil will be investigated. Miniaturization of the measurement system using innovative photonic components, such as those described in [18,19], is going to be the other subject of research at this stage.

In this paper, the variability of the spectral characteristics of mint grown under different lighting conditions was examined. The leaves used in this study come from a small hydroponic plantation of round-leaved mint species (*Mentha rotundifolia*) illuminated by light of several different spectra, treated as the parameter of plant growth. The research project covers measurements of spectral characteristics of leaves, as well as colorimetric analysis. In addition, the results of mass yield measurement are provided as an indication of the existence of a relationship between the spectra of lighting used in cultivation and the contents of the plants.

## 2. Materials and Methods

Research on spectral properties of medicinal plant leaves grown in different lighting condition was performed, based on experimental cultivation. It was organized in a darkened room (without the access to daylight), similar to a vertical farm. Due to the fact that mint grows well in aquatic environments, it was carried out as a hydroponic cultivation [20,21].

A dedicated measurement system was developed for the spectral measurements of leaves. It consists of a high-end spectroradiometer, a translation stage with a leaf holder and an illuminating lamp of broad and continuous spectrum. A high-efficiency lamp is required to provide a sufficient amount of light without overheating the surface of the leaf. Thus, the developed lamp is based on a moderately driven halogen supported by a set of power LEDs [22,23]. To properly mix light from several different LEDs, a dedicated low-cost integration sphere, made of two metal hemispheres (one with an output hole), was applied [24]. To ensure high diffusive reflectance of internal coating of the sphere, the inner surface of the sphere was covered with a custom-formulated paint based on barium sulphate and white latex paint [25,26]. The reflectance of the paint was high (over 90%) and approximately constant over the whole spectrum range of interest. Additionally, to improve the cooling of the leaf, there was an air gap between the illumination sphere and the tested leaf.

**Figure 1:**
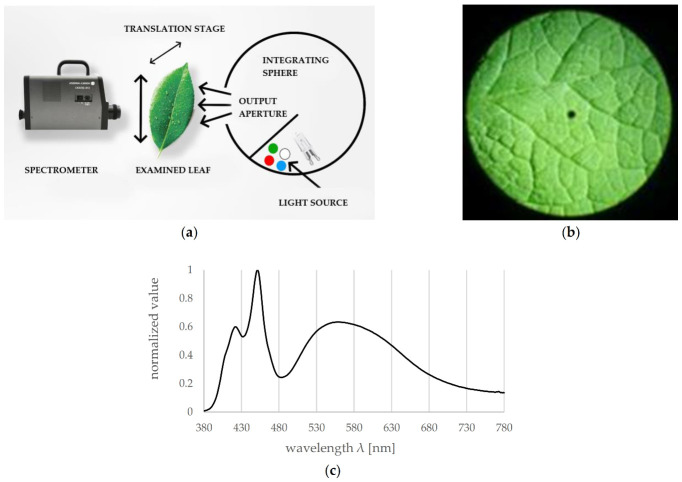
(**a**) Block diagram of the measurement setup. (**b**) View of the tested leaf through the instrument viewfinder; small black dot represents the measurement field. (**c**) Spectrum of the developed illumination source, normalized to its highest intensity.

The block diagram of the developed setup is presented in Figure 1a. A Konica Minolta CS-2000 (Osaka, Japan) spectroradiometer was used to record measured spectra reflected from or transmitted by the tested leaves. A view of a tested leaf through the instrument viewfinder is shown in Figure 1b. In order to account for the spectral characteristics of the light source used in the measurement, a reference spectrum, shown in Figure 1c, was recorded with a diffuser plate used in place of the leaf.

The tissue of leaves is of much larger thickness compared to the wavelength of light and can be considered a highly scattering material. For such materials, the reflected and transmitted light mostly contains an internal diffusion component, dependent on the absorption of the object. Thus, the measurements of reflectance, transmittance and absorbance are equivalent (in terms of the shape of the spectrum). For convenience reasons, the developed measurement system was set up to perform transmissive measurements of the plant leaves.

For convenient determination of amount of light emitted by applied illumination sources, in terms relevant to plants’ growth, the portable spectrometer GL Optic Spectis 1.0 (Puszczykowo, Poland) Touch was used, providing PPF and PPFD (photosynthesis photon flux, photosynthesis photon flux density) quantities commonly used by planters, lamp designers and scientists [27,28]. This device can also measure the spectrum of the light sources.

During the research project presented in this paper, dry mass yield at the end of the cultivation was determined by/with a BTS 110 moisture analyzer. Based on halogen radiators, this analyzer offers the resolution of 10 mg and a humidity determination accuracy of about 1%.

## 3. Experimental

In accordance with the assumptions made, a test cultivation was prepared. In accordance with the adopted assumptions, a test cultivation was prepared. The species of *Mentha Rotundifolia* was selected for cultivation. To provide a mechanical support for roots in the hydroponic plantation, rockwool slabs were used. A total of six cultivation stands were prepared, supplied with water and minerals from one common source using a hydroponic system and a common ventilation system. Each stand housed seven plants and was illuminated by light with a different spectrum. There were 42 mint plants in total.

The performed cultivation lasted 6 weeks in total: 2 weeks from sowing to seedling and 4 weeks from seedling to harvest. The day/night ratio was set to 2:1, i.e., 16 h of light (day) and 8 h of darkness (night) [29]. During the cultivation period, the average environmental parameters were as follows: temperature, 25° C [30], humidity, 50%, carbon dioxide content, 420 ppm. The water supply flow rate was set to about 60 L/h and measured water pH was in the range of 6.3–6.6 [31]. The water nutrient content, measured in an external laboratory, was sodium, 46 (mg/L), potassium, 4 (mg/L), calcium, 120 (mg/L), magnesium, 14 (mg/L), and iron, 14 (ug/L). For the experiment, six lamps with different spectra were selected, one for each of the available six individual cultivation stands. Four of the selected lamps were commercially available, while the other two were (our) custom designs, based on the literature data. Lamp selection was limited to those that allow the proper cultivation of plants. These lamps use a mixed spectrum that is a combination of spectrum of white, blue, red and far-red LEDs. As reported, the ratios of power carried by individual components of the spectrum may affect selected properties of various plant species, such as weight yield [32,33,34], leaf color change [15,16], carotenoid and chlorophyll content [35], vitamin C content [36], grafting [37], or flowering [13,38]. Two major peaks at blue (450 nm) and red (650 nm) wavelength correspond to the absorption characteristics of chlorophyll A and B [12].

**Figure 2:**
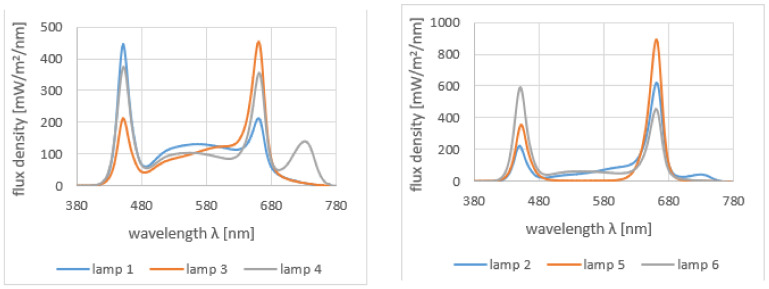
Spectral characteristics of the lamps used in mint cultivation.

Lamps 1, 3 and 6 were based on white, blue and red LEDs, with the power ratio of red to blue light different for each lamp. Lamp 5 utilized only red and blue light, in the recommended [34] ratio of 70:30. Finally, lamps 2 and 4 used white, blue, red and far-red LEDs. The power delivered by far-red LEDs was higher in the latter lamp, as was the blue/red ratio. The spectral characteristics of all the lamps used are shown in Figure 2.

The applied lamps were equipped with drivers that enabled the adjustment of the light intensity. They were driven to ensure the same PPFD (photosynthesis photon flux density) for all lamps, necessary for a comparative analysis. The lamps were mounted at about 200 mm above the cultivation level and PPFD measurements were taken on the cultivation level with the detector placed on the axis of symmetry of the lamps. They were controlled so that the amount (photosynthesis photon flux density) of the radiation useful for plants (PPFD) was the same for all lamps. The measured PPFD values for all six lamps are presented in Table 1. As can be noticed, the PPFD values for the individual lamps are very similar. The differences do not exceed 2%. Taking into account the potential inaccuracy of the positioning of the PPFD meter, the error in setting the PPFD value was estimated at 5%.

## 4. Results and Discussion

Proper growth of mint was observed in all cultivation stands, irrespectively of the illuminating lamp used, including the lamp with a bi-chromatic spectrum corresponding to the chlorophyll absorption bands (lamp 5). At the end of the cultivation, shortly before harvesting, the spectra of mint leaves grown under different lighting conditions were measured. Measurements were made using the measurement system described in the previous Section, with the spectroradiometer measuring angle set to 0.1° [15] and the close-up lens attached. In this configuration the spectrum was acquired from an area of about 0.15 mm in diameter.

**Figure 3:**
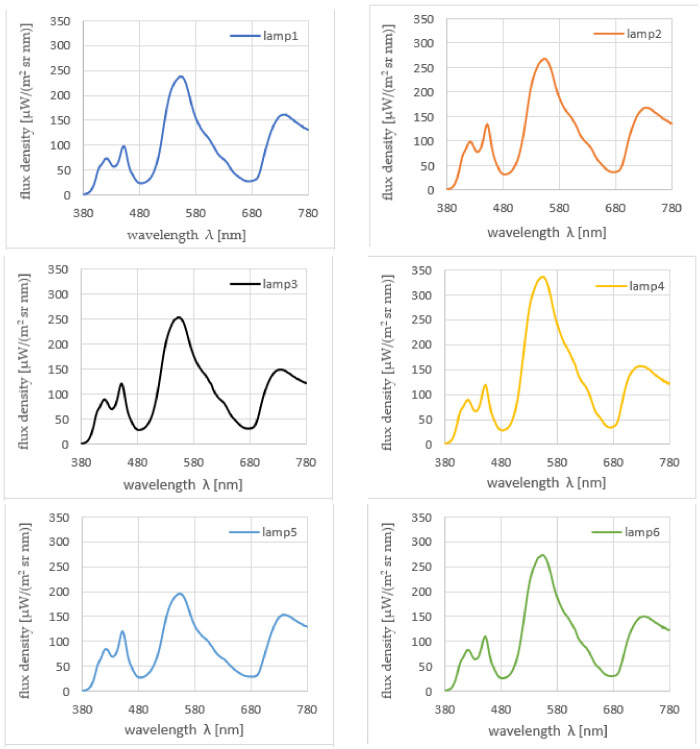
Averaged spectral characteristics of mint leaves grown under the light of all tested lamps.

As the results could vary depending on the point of measurement of the leaf tissue, (e.g., in the leaf vascular system area or outside the leaf vascular system area), the measurements were carried out at different points of the leaf and for various leaves. In total, between 10 and 15 measurements of spectrum were performed on leaves from each cultivation stand. The individual spectra were averaged and presented in Figure 3.

There are differences between the spectra in Figure 3, especially in terms of level, but it is difficult to distinguish individual features of these spectra in terms of shape.

**Figure 4:**
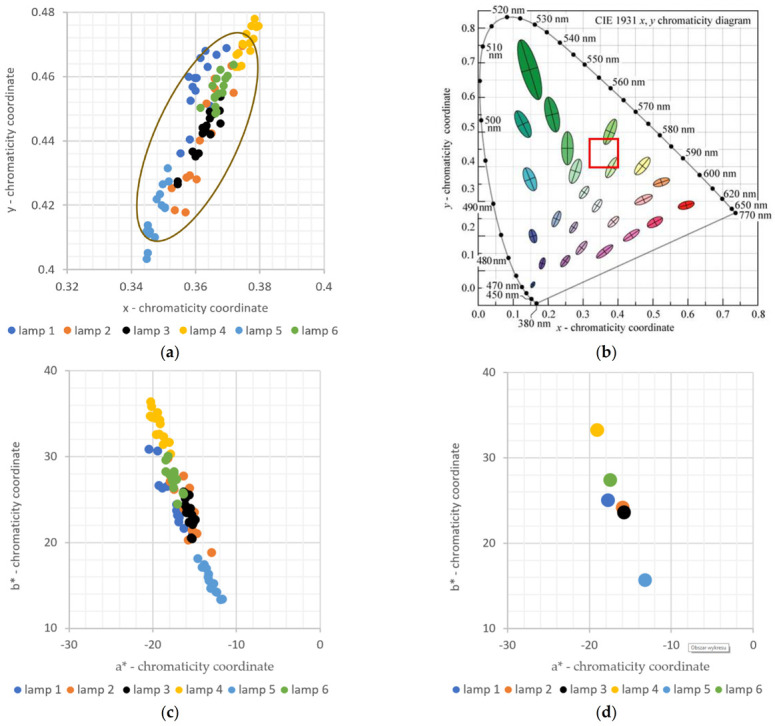
(**a**) CIE 1931 colorimetric coordinates of all measured points of mint leaves, with a MacAdam ellipse, magnified 10× for better readability. (**b**) CIE 1931 colorimetric diagram with marked MacAdam ellipses [39] and the red square marking the area presented in (**a**). (**c**) CIE 2000 L*a*b* colorimetric coordinates of all measured points of mint leaves. (**d**) Averaged CIE 2000 L*a*b* colorimetric coordinates of all measured groups of leaves; MacAdam ellipses in (**b**) are also magnified 10× for better readability.

Investigation of these differences required a spectral analysis technique to be used to provide observer-independent, objective results. After consideration, colorimetric analysis (according to CIE standards) was selected [39]. In the first step of analysis, the points corresponding to the determined chromaticity coordinates x, y for all measured leaves were calculated from the acquired spectra and plotted on the CIE 1931 color diagram [26] (Figure 4a). Despite its perceptual non-uniformity, the CIE 1931 color space is still quite popular. The colors of the points correspond to the lamp under which a given plant was grown. The region of the CIE 1931 color diagram relevant to our study is shown in Figure 4a, with a complete CIE 1931 diagram presented in Figure 4b, with area from Figure 4a marked by the red rectangle.

It is apparent from Figure 4a, that the data points corresponding to leaves grown under the light of individual lamps form distinct clusters. The points within each cluster show dispersion, the amount of which varies from cluster to cluster. There is also a partial overlap among these clusters.

The cluster in the lower middle area of the graph corresponds to lamp 5, the spectrum of which is bi-chromatic, i.e., contains only red and blue colors. In turn, the cluster corresponding to lamp 1 (the spectrum with the highest green content) is shifted up (towards the green region in the CIE color space), while the cluster corresponding to lamp 4 (containing far-red LEDs) is shifted to the right and up (towards the yellow region in the CIE color space). For the remaining lamps (lamp 2, lamp 3 and lamp 6) the color of the leaves is similar and corresponding clusters are located near the center of the group of all data points shown in Figure 4a.

Before discussing the ability of a human observer to discern the colors corresponding to the leaves used in this study, let us recall the boundary of the perception of the color difference, often referred to as just noticeable difference—JND. In the CIE 1931 colorimetric diagram, this boundary has an elliptical shape, often called the MacAdam ellipse [39], that varies in size and orientation depending on the color, as shown in Figure 4b. The ellipses in Figure 4b are enlarged by a factor of 10, to improve readability. A MacAdam ellipse, also enlarged by a factor of 10, was also superimposed on Figure 4a. As can be seen, the colors corresponding to the leaves grown under different lamps are in most cases discernible by a human observer.

However, one of the best metrics for perceptual discrimination of colors is the CIE ΔE parameter, defined for the most perceptual-uniform color space CIE 2000 L*a*b*. Thus, CIE 2000 chromaticity coordinates of all measured leaves were calculated and are presented on the a* -- b* chromaticity plane in Figure 4c. Following, these colorimetric coordinates were averaged within each set of leaves, yielding points shown in Figure 4d. Color differences between the averaged colorimetric coordinates were determined according to CIE 2000 ΔE metric and are presented in Table 2 [40].

Based on the results presented in Table 2, it can be concluded that most color differences are above the ΔE = 2.3 limit, allowing satisfactory discrimination to be performed by a human observer. There are only two exceptions, where ΔE is below the 2.3 limit, and they are for plants grown under lamp 2 and lamp 3 and under lamp 2 and lamp 6.

Moreover, averaged colorimetric coordinates corresponding to each set of leaves are distinguishable when using a spectrophotometer of adequate accuracy.

**Figure 5:**
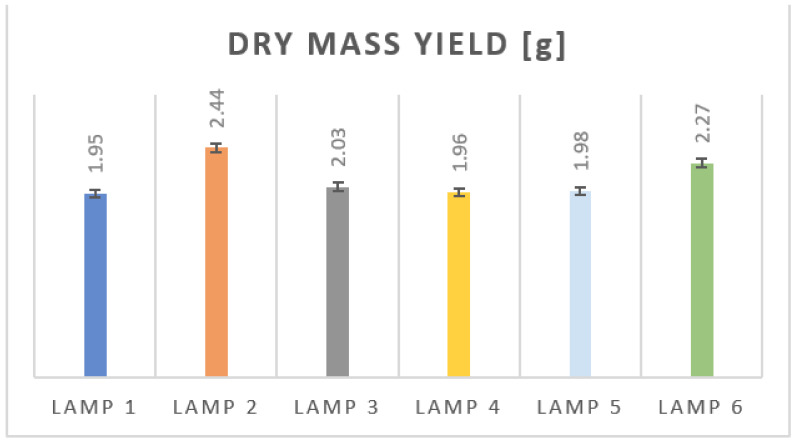
Dry mass yield of the mint harvested. Average value of 7 mint plants was presented for each lighting conditions.

The measurement of dry mass yield of mint plants used in this study and harvested at the end of the cultivation was the second important component of the first research stage (leaf spectrum measurements). All seven plants from each cultivation stand were used. The averaged results are shown in Figure 5, for each lamp, respectively. Total measurement error was estimated to be 1.5%, based on the accuracy of humidity determination (1%) and weight measurement resolution (10 mg).

As can be seen, the highest mass yield was obtained for lamp 2 and lamp 6. These are lamps with the spectrum content in the green range, but kept at a relatively low level. The colorimetric points for the leaves grown under these lamps are located near the center of the group of all determined colorimetric coordinates. This might imply that the leaves that have these particular color coordinates are those in which the mass yield is the highest.

Inconclusive results were obtained from the mass yield for lighting with lamp 3. The colorimetric points for the leaves grown under it are close to the points corresponding to leaves grown under lamps 2 and 6, but the mass yield was lower, only slightly higher than that for the other three lamps, i.e., lamp 1, 4 and 5. Probably, flux density of lamp 3 in the 480–600 nm range was too high in relation to flux at blue (450 nm) and red (650 nm) wavelength ranges. This issue will be addressed during further research.

Based on the presented results, it can be concluded that the chromaticity of leaves depends on the spectrum of light used in the cultivation. Similar dependence is observed for dry mass yield, fulfilling the necessary condition for initiating the second stage of research.

In the second stage, that will be inspired by the results in this work, the relationship between the spectral properties of leaves and chemical composition of mint oil will be investigated, employing biochemical analysis techniques. The research project will be focused on measuring contents of menthol, flavonoids and carotenoids, as well as chlorophyll a and b. The leaves will be processed and individual components of interest will be extracted. Their quantity will be determined by chemical or spectroscopic methods. Another focal area of research at this stage will be tailoring the measurement system to the needs of application using innovative photonic components, such as those described in [18,19], as well as solutions and integration techniques developed and tested in the area of microsystems, lab-on-a-chip, for instance those discussed in [41,42]. Finally, alternative spectral processing techniques, such as the support vector machine, will be considered, if the need for extracting more information from the acquired spectra arises.

## 5. Conclusions

An examination of variability of spectral properties of mint leaves growing in different lighting conditions was carried out. Based on the experiments, the colorimetric technique turned out to be satisfactory for spectrum discrimination. Averaged spectra of leaves from each cultivation stand are distinguishable when using a spectrophotometer and—in most cases—by a human observer. Since the spectrum of lighting can affect the content of medicinal substances and there are noticeable differences in the spectrum of leaves, it is reasonable to carry out the next stage of research relating to the correlation between the spectra of leaves and the content of medicinal substances.

## Figures and Tables

**Table 1:** The measured PPFD values for the applied lamps.

Light Source	Lamp 1	Lamp 2	Lamp 3	Lamp 4	Lamp 5	Lamp 6
PPFD [µmol/m^2^/s]	165.5	166.7	167.6	167.1	167	167.1

**Table 2:** Calculated ΔE CIE2000 between colorimetric points for mint leaves grown under all lamps.

Light Sources	Lamp 1	Lamp 2	Lamp 3	Lamp 4	Lamp 5	Lamp 6
Lamp 1	-	3.55	2.67	8.35	2.94	3.73
Lamp 2	3.55	-	1.03	6.76	8.12	1.88
Lamp 3	2.67	1.03	-	5.10	3.93	2.43
Lamp 4	8.35	6.76	5.10	-	15.37	3.20
Lamp 5	2.94	8.12	3.93	15.37	-	9.90
Lamp 6	3.73	1.88	2.43	3.20	9.90	-

## Data Availability

Not applicable.

## References

[1] Yahia I.B.H., Jaouadi R., Trimech R., Boussaid M., Zaouali Y. (2019). Variation of chemical composition and antioxidant activity of essential oils of *Mentha* x *rotundifolia* (L.) Huds. (*Lamiaceae*) collected from different bioclimatic areas of Tunisia. Biochem. Syst. Ecol..

[2] Grzeszczuk M., Jadczak D. (2009). Estimation of biological value of some species of mint (*Mentha* L.). Herba Pol..

[3] Balakrishnan A. (2015). Therapeutic Uses of Peppermint—A Review. J. Pharm. Sci. Res..

[4] Derwich E., Benziane Z., Taouil R., Senhaji O., Touzani M. (2010). Aromatic plants in Morocco: GC/MS analysis of essential oils of leaves of Mentha piperita. Adv. Environ. Biol..

[5] Eteghad S.S., Mirzaei M., Pour S.F., Kahnamui S. (2009). Inhibitory Effects of endemic Thymus vulgaris and Mentha piperita essential oils on *Escherichia coli* O157:H7. Res. J. Biol. Sci..

[6] Kizil S., Hasimi N., Tolan V., Kilinc E. (2010). Mineral content, essential oil components and biological activity of two mentha species (*M. piperita* L., *M. spicata* L.). Turk. J. Field Crops.

[7] Kałwa K., Wilczyński K., Olesińska K. (2017). Wpływ warunków przechowywania suszonej Mięty pieprzowej (*Mentha piperita* L.) na antyoksydacyjne właściwości otrzymanych naparów oraz zawartość i skład olejku eterycznego (The effect of storage conditions of dried Peppermint *Mentha piperita* L. on the antioxidant properties of the infusions obtained and the content and composition of essential oils. Acta Sci. Pol. Tech. Agrar..

[8] Canter P.H., Thomas H., Ernst E. (2005). Bringing medicinal plants into cultivation: Opportunities and challenges for biotechnology. Trends Biotechnol..

[9] Fukuda N. (2019). Plant Growth and Physiological Responses to Light Conditions. Plant Factory Using Artificial Light.

[10] Cegielski T., Bujalski D., Kowalczyk K., Gajc-Wolska J., Hemka L. (2016). Use of light emission programming in tomato grow light system. Proc. Electrotech. Inst..

[11] Nelson J.A., Bugbee B. (2014). Economic Analysis of Greenhouse Lighting: Light Emitting Diodes vs. High Intensity Discharge Fixtures. PLoS ONE.

[12] MacCree K.J. (1972). Test of current definitions of photosynthetically active radiation against leaf photosynthesis data. Agric. Meteorol..

[13] Faline D.M., Plantenga S.W., Bergonzi C., Bachem W.B. (2019). High light accelerates potato flowering independently of the FT-like flowering signal StSP3D. Environ. Exp. Bot..

[14] Christiaensac A., Gobina B., Huylenbroeckb J.V. (2019). Adventitious rooting of Chrysanthemum is stimulated by a low-red:far-red ratio. J. Plant Physiol..

[15] Meng M., Runkle E.S. (2020). Growth Responses of Red-Leaf Lettuce to Temporal Spectral Changes. Front. Plant Sci..

[16] Ohtake N., Ishikura M., Suzuki H., Yamori W., Goto E. (2018). Continuous Irradiation with Alternating Red and Blue Light Enhances Plant Growth While Keeping Nutritional Quality in Lettuce. Hort. Sci..

[17] Magagnini G., Grassi G., Kotiranta S. (2018). The Effect of Light Spectrum on the Morphology and Cannabinoid Content of *Cannabis sativa* L.. Med. Cannabis Cannabinoids.

[18] Xu K. (2021). Silicon electro-optic micro-modulator fabricated in standard CMOS technology as components for all silicon monolithic integrated optoelectronic systems. Micromech. Microeng..

[19] Xu K., Chen Y., Okhai T.A., Snyman L.W. (2019). Micro optical sensors based on avalanching silicon light- emitting devices monolithically integrated on chips. Opt. Mat. Expr..

[20] Kozai T., Niu G., Takagaki M. (2020). Plant Factory. An Indoor Vertical Farming System for Efficient Quality Food Production.

[21] Hoang H.N., Kitaya Y., Shibuya T., Endo R. (2019). Development of an in vitro hydroponic culture system for wasabi nursery plant production—Effects of nutrient concentration and supporting material on plantlet growth. Sci. Hortic..

[22] Mottier P. (2009). LEDs for Lighting Applications.

[23] DiLaura D.L., Houser K.W., Mistrick R.G., Steffy G.R. (2011). The Lighting Handbook.

[24] (2007). Measurement of LEDs.

[25] Mazikowski A., Feldzensztajn M. (2017). Lamp of adjustable spectrum for photographic usage. Proc. SPIE.

[26] Kington N., Bugbee B. (2005). A Mixture of Barilium Sulfateand White Paint is a Low-Cost Substitute Reflectance Standard for Spectralon, Utah State University. http://www.triticeaecap.org/wp-content/uploads/2011/12/Barium_Sulfate.pdf.

[27] Chen X.L., Wang L.C., Li T., Yang Q.C., Guo W.Z. (2019). Sugar accumulation and growth of lettuce exposed to different lighting modes of red and blue LED light. Sci. Rep..

[28] Nanya K., Ishigami Y., Hikosaka S., Goto E. (2012). Effects of blue and red light on stem elongation and flowering of tomato seedlings. Acta Hortic..

[29] Inoue F., Sugiura H., Tabuchi A., Karasawa D., Minami M. (2003). Plant Regeneration of Peppermint, Mentha plperlta from the Hairy Roots Generated from Microsegment Infected with Agrobacterium rhizogenes. Plant Biotechnol..

[30] Islam M., Dembele D., Keller E.R. (2005). Influence of explant, temperature and different culture vessels on in vitro culture for germplasm maintenance of four mint accessions. Plant Cell Tissue Organ Cult..

[31] Nozzi V., Graber A., Schmautz Z., Mathis A., Junge R. (2018). Nutrient Management in Aquaponics: Comparison of Three Approaches for Cultivating Lettuce, Mint and Mushroom Herb. Agronomy.

[32] Litvin A.G., Wilson L.A., Currey C.J. (2020). Effects of supplemental light source on basil, dill, and parsley growth, morphology, aroma, and flavor. J. Am. Soc. Horticult. Sci..

[33] Joshi N.C., Ratner K., Eidelman O., Bednarczyk D., Zur N., Many Y., Shahak Y., Aviv-Sharon E., Achiam M., Gilad Z. (2019). Effects of daytime intra-canopy LED illumination on photosynthesis and productivity of bell pepper grown in protected cultivation. Sci. Hortic..

[34] Metallo R.M., Kopsell D.A., Sams C.E., Bumgarner N.R. (2018). Influence of blue/red vs. white LED light treatments on biomass, shoot morphology, and quality parameters of hydroponically grown kale. Sci. Hortic..

[35] Frede K., Schreiner M., Baldermann S. (2019). Light quality-induced changes of carotenoid composition in pak choi *Brassica rapa* ssp. Chinensis. J. Photochem. Photobiol. B Biol..

[36] Gautier H., Rocci A., Buret M., Grasselly D., Dumas Y., Causse M. (2005). Effect of photoselective filters on the physical and chemical traits of vine-ripened tomato fruits. Can. J. Plant Sci..

[37] Singh H., Kumar P., Chaudhari S., Edelstein M. (2017). Tomato Grafting: A Global Perspective. HortSci. Horts.

[38] Croser J.S., Pazos-Navarro M., Bennett R.G., Tschirren S., Edwards K., Erskine W., Creasy R., Ribalta F.M. (2016). Time to flowering of temperate pulses in vivo and generation turnover in vivo–in vitro of narrow-leaf lupin accelerated by low red to far-red ratio and high intensity in the far-red region. Plant Cell Tiss Organ Cult..

[39] Haining R.R., Bimber O. (2011). Displays.

[40] Luo M.R., Cui G., Rigg B. (2001). The Development of the CIE 2000 Colour-Difference Formula: CIEDE2000. Color Res. Appl..

[41] Niculescu A.-G., Chircov C., Bîrcă A.C., Grumezescu A.M. (2021). Fabrication and Applications of Microfluidic Devices: A Review. Int. J. Mol. Sci..

[42] Mejía-Salazar J.R., Rodrigues Cruz K., Materón Vásques E.M., Novais de Oliveira O. (2020). Microfluidic Point-of-Care Devices: New Trends and Future Prospects for eHealth Diagnostics. Sensors.

